# Missing of intervention in children orthopedics - benefits or losses? Presentation of pilot project results

**DOI:** 10.1186/1878-5085-5-S1-A162

**Published:** 2014-02-11

**Authors:** Zbigniew Pilecki, Grzegorz Pilecki, Rostyslav V Bubnov, Halina Kutaj-Wąsikowska

**Affiliations:** 1 Department of Pediatric Orthopedics & Traumatology, Chorzów, Poland; 2 Clinical Hospital ”Pheophania“ of State Affairs Department, Kyiv, Ukraine; 3 National Center For Quality Assessment In Healthcare, Kraków, Poland

## Introduction

The adequate and in time individual intervention planning is fundament of *personalized* treatment, that has to be as minimally invasive as possible. However, refusal from surgery in particular cases may cause serious complications. This part of healthcare was not studied enough, especially in orthopedics. Analysis the causes of resigning from surgery may have strong *predictive* value for children supposed for interventional treatment and become the basis of *preventive* programs for minimizing of intervention.

## The aim

was to analyze the causes of refusal from surgery by children, qualified for orthopedic interventional treatment.

## Material and method

According to the aim we analyzed selected documentation on following areas of healthcare:

1. Among 5,689 children, treated in orthopedic ambulatory in 2012-2013, we separated the group with orthopedic diagnosis codes that required surgical treatment. We distinguished documents of 16 children with initial qualification for surgery.

2. We noticed that 48 children per year weren’t considered for surgery for various reasons: illness of a child, poor school results, rehabilitation or pharmacotherapy carried out, special events, additional testing, the lack of a legal guardian, no telephone contact, resignation from treatment, choosing another institution, etc.

3. Among 1,287 children, treated in the pediatric orthopedic department, 32 were hospitalized due to diagnostic and therapeutic reasons (punctures, biopsies, cuttings taken, blood cultures, diagnostic imaging). Afterwards a separated group of 4 children requiring treatment was distinguished.

4. We analyzed a group of 13 children who stayed for operation and underwent the first stage of treatment and were waiting for the next and also included children, temporarily disqualified from surgery due to illness.

The study was conducted based on the database of patients, treated in the pediatric orthopedic department and ambulatory in 2012-2013 with institution approval, survey was completed on 30.04.2013.

## Results

After formation of first group by the adopted scheme 16 children with their parents were asked to be re-examined. Four children were directed to surgery in our institution (two patients were considered for treatment at the facility for adults - during this period they were aged over 18 years). Second group was the most numerous and complex. Seven children were sent back to the children department of the hospital (one to orthopedic department for adults). In the third group one child was referred for treatment in children department. Formation of fourth groups of three children were directed to hospital treatment in our unit (one to orthopedic department for adults). All were assessed as adverse events that did not cause serious health disorders of patients.

## Conclusion

This testing study, based on subjective scale, shows that health care system fails to provide medical care at sufficient level to many children. Based on the obtained results appropriate algorithm can be adopted for the patient care surveys.

## Outlook and expert recommendations

In the future we plan to introduce such a test as an ongoing process in all hospital departments based on personalized radiology and laboratory medicine. The achieved results allow for recommendation the presented system for surgical practice.

